# Self‐Supervised Deep Learning Framework for Rician Distribution Based Denoising and Modeling of Multi‐b Prostate Diffusion MRI

**DOI:** 10.1002/mrm.70373

**Published:** 2026-04-19

**Authors:** Mustafa Abbas, Wenyin Zhou, Stephan E. Maier, Fredrik Langkilde, Stefan Kuczera

**Affiliations:** ^1^ Department of Radiology, Institute of Clinical Sciences, Sahlgrenska Academy University of Gothenburg Gothenburg Sweden; ^2^ Department of Intelligent Systems School of Electrical Engineering and Computer Science, KTH Royal Institute of Technology Stockholm Sweden; ^3^ Department of Radiology, Brigham Women's Hospital Harvard Medical School Boston MA USA

**Keywords:** ADC, denoising, diffusion, prostate, Rician bias correction

## Abstract

**Purpose:**

Convolutional neural networks (CNNs) are evaluated for improved and accelerated denoising and Rician bias correction in multi‐b DW images with simultaneous signal modeling.

**Methods:**

Prostate diffusion images from 46 individuals acquired at 20 linearly distributed b‐values ($b_{\max}=2000\;s/{\text{mm}}^{2})$ without averaging were used for self‐supervised CNN training. CNNs were trained to output model parameter maps, from which DW images and ADC maps were synthesized, and to account for Rician bias. CNN architectures included were: conventional U‐Net, Attention U‐Net, and Residual Attention U‐Net with biexponential, kurtosis, and gamma distribution as signal models. Moreover, approaches without Rician bias correction and with noise maps as additional input were explored.

**Results:**

For all signal models, synthetic DW images generated from CNN output are of similar quality compared to OBSIDIAN, a model‐based method employing iterative pixel‐wise fitting. Computed ADC and parameter maps obtained with CNN models were less noisy than those obtained with OBSIDIAN. Good quantitative agreement with OBSIDIAN in ROI‐averaged ADC values and, to a slightly lesser extent, kurtosis model parameters was observed. Results with omitted Rician bias correction clearly deviate, while the impact of the noise map input for the CNNs is less pronounced. Computation time was reduced to seconds compared to several hours for OBSIDIAN.

**Conclusion:**

The CNN‐based method has potential in clinical use for achieving higher quality DW images and biomarker maps with drastically reduced computation time. Further refinement and larger training datasets are needed to achieve better generalizability and increased robustness.

## 1 Introduction

The distinct microstructure of healthy and cancerous tissue gives rise to different diffusion properties, which in many cases can be distinguished by the apparent diffusion coefficient (ADC) [1, 2]. In clinical practice, a low and a high b‐value image are acquired, typically b=50–100 and 800–$1000\;{\text{s/mm}}^{2}$, for the computation of an ADC map by employing a monoexponential signal model [3, 4]. Due to the low signal intensity at higher diffusion weightings, signal averaging is used to attain ADC maps with sufficient SNR. This leads to an increase in acquisition time. Although b‐values of 800–$1000\;s/{\text{mm}}^{2}$ are widely applied in prostate imaging, as well as in conditions such as ischemic stroke, several studies indicate that higher diffusion weightings yield improved contrast in diffusion‐weighted images [5, 6, 7]. Therefore, either additional measurements are necessary, prolonging acquisition time or computed DWI needs to be employed, which might result in images with inferior image quality.

Generally, however, due to the complex microstructure in cellular environments, the signal decay as a function of the diffusion weighting b deviates from the simple monoexponential model, which is only correct for Gaussian diffusion as in free water [8]. As a consequence, ADC depends on acquisition parameters such as the maximum diffusion weighting [9, 10]. Higher order models are often used to account for the more complex diffusion behavior [11]. For example, diffusion kurtosis imaging (DKI) uses a second‐order Taylor expansion exponent in the monoexponential model, which incorporates the non‐Gaussian property of diffusion in tissues characterized by the apparent kurtosis K [12, 13]. Higher‐order modelling of the MR signal necessitates the acquisition of a larger number of diffusion weightings. Given the time constraints for a clinical MR exam, using the same amount of averaging for all b‐values is not practical. Furthermore, the computed model parameters, such as ADC, are dependent on the noise level due to the Rician nature of the signal for magnitude MR images [9, 10]. Therefore, other methods for boosting SNR and correcting for Rician signal bias need to be employed. A commonly employed model‐free technique is MP‐PCA [14], where principal component analysis (PCA) is applied for noise reduction with subsequent Rician bias correction [15]. A model‐driven approach that combines denoising, Rician bias correction, and accurate parameter estimation is OBSIDIAN, where iterative pixel‐wise fitting is employed [16]. OBSIDIAN has a slight advantage in computational speed compared to maximum likelihood estimation (MLE), another model‐driven approach [16, 17].

The prostate gland is a prime example where Rician bias due to low SNR affects quantification of ADC and other biomarkers [16, 18]. Low SNR results from the relatively large distance between the gland and the receiver coil setup. In principle, endorectal coils can boost SNR. However, they have their own shortcomings such as time‐consuming preparation and an unpleasant scanning experience for the patient [19]. Moreover, similar complications arise, for example, in brain imaging, where central parts of the brain suffer from low SNR due to receiver coil distance.

Recently, there has been growing interest in deep learning (DL) based denoising and signal modeling methods. Compared to more conventional methods, DL based methods offer very fast computation times [20]. Specifically, convolutional neural networks (CNNs) have seen use in many image‐based tasks and constitute the fundamental structure of other, more advanced models. In one study by Cochran et al. [21], a CNN‐based commercialized DL reconstruction algorithm reduced the noise in prostate diffusion‐weighted images, resulting in higher SNR compared to the conventional reconstruction, allowing for shorter scan times with reduced NSA. Pfaff et al. [22] presented a CNN‐based self‐supervised approach with Stein's unbiased risk estimator for denoising diffusion‐weighted images of the prostate by incorporating noise maps. Generally, self‐supervision has been employed in numerous studies recently for improved resolution and SNR in DWI [23, 24, 25, 26]. Vasylechko et al. [27] implemented a self‐supervised CNN approach for estimating model parameters with the intravoxel incoherent motion (IVIM) model. A study by Tian et al. [28] used a modified self‐supervised U‐Net model to denoise multiple diffusion‐weighted images of low quality, synthesized from tensor fitting based on six acquired diffusion‐weighted images. However, only in very recent work has the Rician nature of the diffusion MRI signal been considered in deep learning‐based estimation of model parameters [29, 30, 31].

Expanding on our previous work [16, 18], where a large number of b‐values are probed, we have implemented several types of CNNs for simultaneous denoising, Rician bias correction, and model parameter estimation for diffusion‐weighted images. Our approach is similar to Vasylechko et al. [27], however, with the addition of incorporating maps on spatially varying noise, crucial for Rician bias correction. This addition also distinguishes our method from the aforementioned work with Rician‐aware parameter estimation [29, 30, 31] where spatial noise maps are not part of the Rician bias correction scheme. Additionally, two of these studies [29, 30] only consider b‐values up to $900\;s/{\text{mm}}^{2}$ less prone to Rician bias in constrast to $2000\;s/{\text{mm}}^{2}$ in our case. Specifically, our algorithms rely on noise maps generated by OBSIDIAN during the training stage for estimation of noise and Rician bias. For inference, however, the computationally intensive noise maps from OBSIDIAN are not needed, allowing for fast image and parameter map generation. It is shown that the denoised images and computed model parameters from the various DL algorithms are comparable to OBSIDIAN results, rendering our framework more feasible in a clinical setting [18].

## 2 Methods

### 2.1 Patient Population

All patients involved in this study participated in the Göteborg Prostate Cancer Screening 2 Trial (G2‐trial) [32]. This trial has been approved by the Regional Ethics Review Board, and all participants in this study provided written consent to be included in the G2‐study. As part of the trial, all G2‐patients with PSA results indicative of suspected cancer underwent diffusion MR imaging in Sahlgrenska University Hospital. Consequently, PSA‐level and Prostate Imaging–Reporting and Data System (PI‐RADS) v2.1 score were available for each participant. This study adopts a dataset comprising 25 patients from our previous study [18] (“Set 1”) together with more recently acquired data from another 25 patients on a different scanner (“Set 2”). An overview of the participant population with their PI‐RADS score is shown in Table 1.

**TABLE 1:** Overview of PI‐RADS scores in the training‐validation and test datasets from two scanners.

Dataset	Category	Training‐validation set	Test set	Total
	Healthy (PI‐RADS < 3)	19	0	19
Set 1	Cancer Patient (PI‐RADS = 3)	1	1	2
	Cancer Patient (PI‐RADS > 3)	3	1	4
	Healthy (PI‐RADS < 3)	20	2	22
Set 2	Cancer Patient (PI‐RADS = 3)	1	0	1
	Cancer Patient (PI‐RADS > 3)	2	0	2
Overall Total		**46**	**4**	50

### 2.2 MRI Acquisition

All patients underwent an examination with a standard clinical prostate MRI protocol on a 3T scanner (Set 1: Philips Achieva dStream, Philips Healthcare; Set 2: Philips MR 7700, Philips Healthcare). A pelvic‐phased array coil, together with the integrated table array coil, was used for signal reception. The imaging protocol for the DL‐based approach, referred to as “21b”, used 21 linearly distributed b‐values ranging from 0 to 2000 s/mm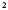 for three orthogonal diffusion directions along the magnet's main axes, but applied the same image geometry as the corresponding clinical protocol. For Set 1, a different gradient mode (called “enhanced” mode by the vendor) was employed for the 21b sequence to attain a higher maximum gradient amplitude at a reduced slew rate. Moreover, the option to use all gradient coils simultaneously for diffusion encoding (called “gradient overplus” by the vendor), resulting in a higher maximum attainable gradient amplitude, had to be turned off to allow images from individual diffusion encoding directions to be exported. For Set 2, the DWI sequence of the standard clinical prostate protocol had a much shorter TE (59ms) than for Set 1, as the scanner was equipped with a more powerful gradient system. In order to compare DW images and ADC maps at similar TE, which has an effect on image contrast and ADC values [33], we opted to employ an additional DWI sequence for the two test set patients in Set 2 with the TE prolonged to match the TE of the 21b sequence for Set 2, which has a TE similar to the sequences in Set 1. Moreover, gradient overplus was deactivated for this sequence. In the following, this sequence is referred to as the clinical sequence for Set 2. The 21b sequence for Set 2 employed gradient‐enhanced mode and no gradient overplus, resulting in slightly shorter TE due to the higher attainable slew rate in comparison to Set 1. In Table 2, acquisition parameters for all sequences are listed. Furthermore, the clinical diffusion‐weighted images in Set 2 at b=1500 s/mm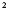 were computed by means of cDWI by the scanner software [34, 35].

**TABLE 2:** Acquistion parameters of 21b and clinical sequences.

Parameter	Set 1 – 21b	Set 1 – Clinical	Set 2 – 21b	Set 2 – Clinical
Scanner	Achieva dStream	Achieva dStream	MR 7700	MR 7700
Acquisition sequence	ss‐EPI	ss‐EPI	ss‐EPI	ss‐EPI
Number of b‐values	21	4	21	3
Acquired b‐values [s/mm 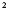 ]	0–2000	0, 100, 1000 & 1500	0–2000	0,100 & 1000
NSA	1	6	1	2, 2 & 14
TE [ms]	77	78	74	74 (59)
TR [ms]	4000	4000	4300	4307 (4000)
Acquisition matrix size	92×77	92×77	92×77	92×77
Acquisition resolution [mm]	3×3	3×3	3×3	3×3
Slice thickness [mm]	3	3	3	3
Slice gap [mm]	0.3	0.3	0	0
FOV [mm]	280×233	280×233	280×233	280×233
Reconstructed voxel size [mm]	1.17×1.17×3	1.17×1.17×3	1.46×1.46×3	1.46×1.46×3
Number of slices	22	22	25	25
Number of diffusion encoding directions	3	3	3	3
Gradient enhanced mode	yes	no	yes	yes
Maximum attainable slew rate [mT/m/ms]	100	200	150	150
Maximum attainable gradient amplitude per coil [mT/m]	62	30	60	60
Gradient overplus	no	yes	no	no (yes)
Maximum attainable gradient amplitude overplus [mT/m]	—	45	—	‐ (88)
Diffusion gradient timing: δ [ms]	15	18	15	15 (8)
Diffusion gradient timing: Δ [ms]	37	38	37	37 (30)
Acquisition duration [s]	252	244	271	220 (204)

*Note*: NSA applies to all b‐values except for Set 2‐Clincal where NSA is listed individually from lowest to highest b‐value. With the gradient‐enhanced mode, a higher maximum gradient amplitude is attainable at the cost of a reduced slew rate. With the gradient overplus option activated, all gradient coils are used simultaneously for diffusion encoding to attain a higher total gradient amplitude. Maximum attainable gradient amplitude overplus refers to the maximum attainable gradient amplitude in the case that gradient overplus is activated. Clinical sequence in Set 2 refers to an additional DWI sequence performed for the two patients in the Set 2 test set; if different from the additional DWI sequence, values for the standard clinical DWI sequence run for all patients in Set 2 are provided in parentheses.

Parameters common to all protocols: Phase encoding direction: AP; fat suppression: SPAIR; two‐fold coil acceleration and vendor‐supplied 2D geometric distortion correction applied.

### 2.3 Higher‐Order Model Functions

The clinically used apparent diffusion coefficient (ADC) is based on the monoexponential model that describes the water diffusion signal S(b) as:

(1) $$S(b)=S_{0}e^{-b\text{ADC}},$$

where $S_{0}$ denotes the signal acquired without diffusion weighting. However, as previously mentioned, in complex microstructures, the diffusion signal does not follow a monoexponential decay. Various models have been employed with the aim of more accurately capturing the signal behavior. The biexponential model splits the diffusion signal in tissue into two components

(2) $$S(b)=S_{0}\left(fe^{-bD_{f}}+(1-f)e^{-bD_{s}}\right),$$

where f is the signal fraction of a fast diffusion compartment with diffusion coefficient $D_{f}$, while $D_{s}$ is the diffusion coefficient of a slow diffusion compartment. The kurtosis model

(3) $$S(b)=S_{0}e^{-b{\text{ADC}}_{K}+\frac{1}{6}b^{2}{\text{ADC}}_{K}^{2}K},$$

is a second‐order Taylor expansion in the exponent of the monoexponential model, where the diffusion is characterized by the kurtosis apparent diffusion coefficient ${\text{ADC}}_{K}$ and by which the deviation from a Gaussian distribution is described with the kurtosis parameter K. The gamma distribution model, described by

(4) $$S(b)=S_{0}{\left(1+\theta \cdot b\right)}^{-k},$$

whereby k and θ are the shape and scale parameters of the gamma distribution, is a statistical approach to describe signal attenuation with b‐value [36]. In contrast to the biexponential model, the gamma distribution model assumes the diffusion coefficient to be continuously distributed rather than represented by discrete coefficient values. Here, the biexponential, kurtosis, and gamma distribution model were used to describe the signal as part of the DL‐based reconstruction. As these models do not include any directional dependence, each diffusion direction was modeled independently, except for $S_{0}$, which was shared over all directions [16, 18]. Hence, the signal equation can be expressed as a 3D vector $S(b)=[S_{M}(b),S_{S}(b),S_{P}(b)]$ where the aforementioned models $S_{i}(b)$ were ensembled from each direction i={M,S,P}, but only with a single $S_{0}$ parameter. For example, in the case of the kurtosis function, this results in 7 independent model parameters.

### 2.4 Data Preprocessing

Images from four patients were chosen as testing data (see Table 1). The remaining image data from 46 patients were split into training and validation sets, with 20% of the images used for validation. A five‐fold cross‐validation was implemented, ensuring that all samples were included in the validation set once across all folds. For 3D signal models, each data sample corresponds to a single image slice of the 21b scan and consists of 60 images acquired across 20 distinct b‐values (100 to 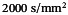) for each diffusion encoding direction. The $b_{0}$‐image was excluded to avoid perfusion related effects [18]. To ensure the same voxel size, DW‐images from Set 2 were interpolated to 240×200 using the TorchIO toolkit [37]. Furthermore, OBSIDIAN and direct model fitting (referred to as “direct fit”) was applied to the 21b patient data, also with the $b_{0}$‐image excluded. While the results for the test cases were used for algorithm comparison, the noise maps, that is, maps of the underlying Gaussian noise standard deviation $\sigma_{g}$, computed by OBSIDIAN for the training cases, were needed for the DL algorithm. Finally, each data sample with its respective noise map was individually normalized with a single scalar, defined as the maximum pixel value observed across the 60 diffusion images. Preprocessing was identical for training and inference.

### 2.5 Deep Learning Models

The following CNNs were implemented: standard U‐Net, Attention‐based U‐Net, and Residual Attention U‐Net. Their architectures are illustrated in Figure S7. The standard U‐Net is a well‐known neural network, first introduced by Ronneberger et al. [38], that is widely‐used for image segmentation and is effective in parameter estimation [39]. The standard U‐Net is based on a series of CNN layers and has a U‐shaped architecture consisting of the so‐called encoder and decoder. Spatial features are extracted in the encoder, which then, together with spatial information from skip connections, are used by the decoder to reconstruct an image through transpose convolution. A variant of the U‐Net architectures is the Attention U‐Net, which implements so‐called attention gates in the skip connections [40]. It outputs weighting matrices that are multiplied by feature maps from the encoder, which enables the neural network to learn, giving more weight to the more relevant regions. Another variant is the Residual Attention U‐Net, which implements residual connections in the backbone of the U‐Net architecture, which helps with stabilizing the training and avoiding vanishing gradients [41, 42].

### 2.6 Deep Learning Algorithm

**FIGURE 1:**
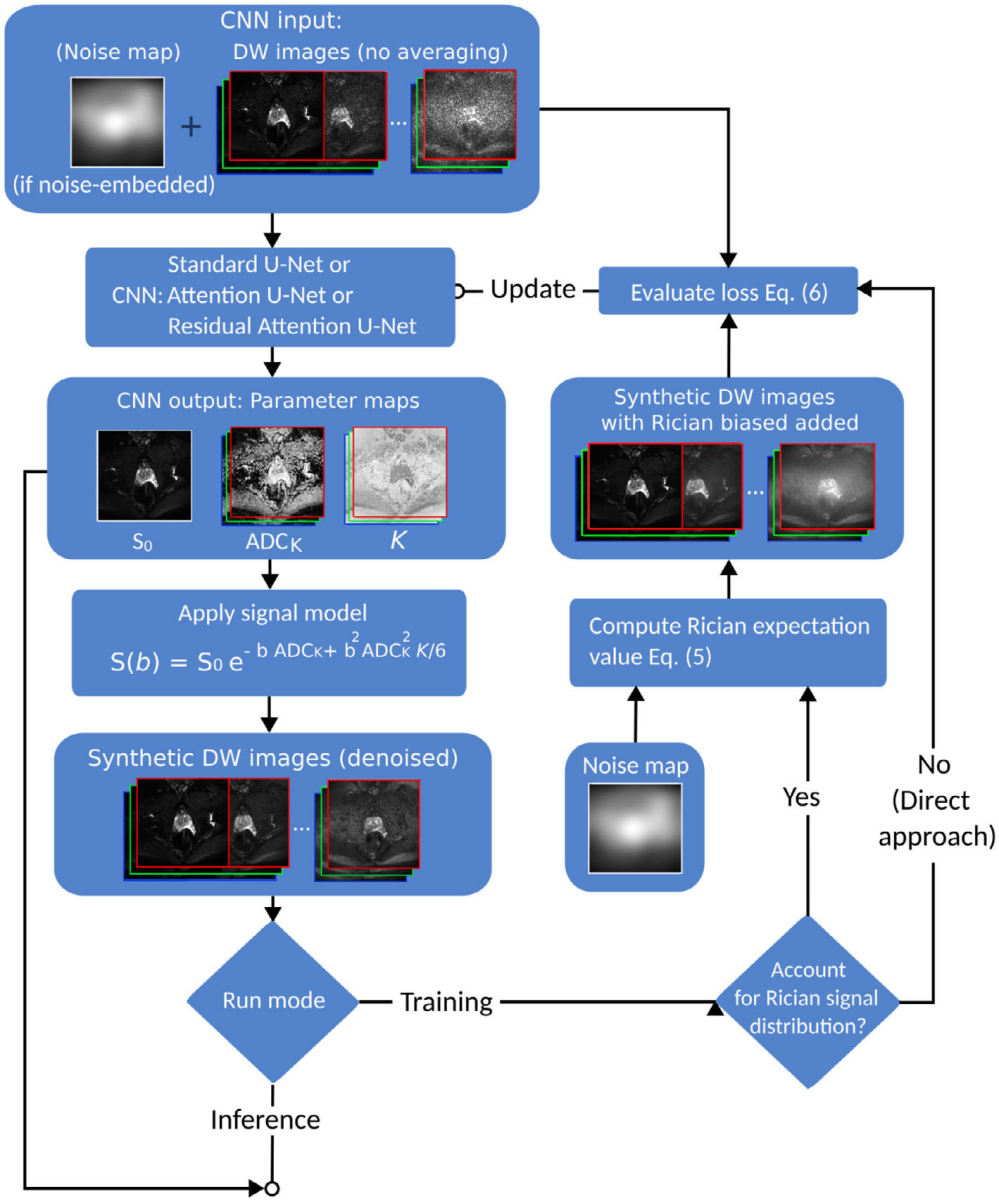
Schematic diagram illustrating the network training cycle and the final output during inference of trained models with the kurtosis model function (Equation 3) as an example. Diffusion‐weighted images, acquired without signal averaging at 20 linearly distributed b‐values ranging from 100 to $2000\;{\text{s/mm}}^{2}$ for three diffusion encoding directions M, P and S indicated by different frame colors were input to the CNN. The parameter maps provided by the CNN were used to generate synthetic diffusion‐weighted images. For Rician bias‐aware CNNs, Rician bias is added to the images using noise maps computed by OBSIDIAN before loss calculation. In the final step, the loss between the CNN input and the synthetic DW images is evaluated to adjust the CNN's parameters. For inference the synthetic diffusion‐weighted images and the parameters are the output, hence no noise map is needed. The “noise‐embedded” approach, however, includes the noise map as an additional input to the CNN.

The implemented DL algorithm is depicted in Figure 1. The code for the algorithm can be found at https://github.com/dMRI‐GU/DL‐OBS. For self‐supervised training, the acquired diffusion‐weighted images were used both as input to the CNN and the loss function. The output of the CNNs are parameter maps of the respective higher‐order model function. From the parameter maps, synthetically generated pixel values are computed by applying the same model function. Subsequently, for each pixel i, the expectation value ${\bar{S}}^{\left(i\right)}=\langle M(S^{\left(i\right)},\sigma_{g}^{\left(i\right)})\rangle$ of the Rician distributed random variable M was calculated, where

(5) $$\begin{array}{ll} \left\langle M(S,\sigma_{g})\right\rangle & =\sigma_{g}\sqrt{\frac{\pi }{8}}\exp\left(\frac{S^{2}}{4\sigma_{g}^{2}}\right)\\ & \;\times \left[\left(\frac{S^{2}}{\sigma_{g}^{2}}+2\right)I_{0}\left(\frac{S^{2}}{4\sigma_{g}^{2}}\right)+\frac{S^{2}}{\sigma_{g}^{2}}I_{1}\left(\frac{S^{2}}{4\sigma_{g}^{2}}\right)\right]. \end{array}$$

The pixel standard deviation $\sigma_{g}^{\left(i\right)}$ was taken from the OBSIDIAN‐generated noise maps and $S^{\left(i\right)}$ is the synthetically generated pixel value. As such, the synthetic pixel vaules $S^{\left(i\right)}$ are treated as temporary true signals in each optimization iteration. Consequently, this operation results in synthetic images with added Rician bias and pixel values ${\bar{S}}^{\left(i\right)}$.

The synthetic images with added Rician bias are then compared to the noisy input images on a pixel‐wise basis by means of a loss function. The loss function L(x,y) is defined as the mean absolute error (MAE) between the input x and output diffusion‐weighted images y:

(6) $$L(x,y)=L_{1}(x,y),\text{with}\;L_{1}=\overset{N}{\underset{n=1}{\sum }}\frac{\left|x_{n}-y_{n}\right|}{N},$$

where N includes every image pixel across all b‐values and diffusion encoding directions. The network architectures standard U‐Net, Attention U‐Net and Residual Attention U‐Net, were trained for three signal models, namely biexponential (2) kurtosis (3) and gamma distribution (4) with the $L_{1}$‐loss function (6). The standard U‐Net model was also trained without added Rician bias, that is, assuming a Gaussian signal distribution, serving as an equivalent to direct model fitting. This approach is referred to as “Standard U‐Net (direct)”. For the kurtosis model function, Residual Attention U‐Net was also trained with the noise map included as additional input aside the diffusion‐weighted images to study the effect of noise information on image and parameter map output. This method hereafter is referred to as noise‐embedded framework.

The models were constrained to estimate parameter maps within defined ranges by applying a sigmoid function to the final layers. Those ranges were equivalent to the parameter boundaries used for OBSIDIAN and direct fit (see Supporting information in Kuczera et al. [16]). The only exception was the kurtosis function were the K parameter range was set to [0,5] in all cases in order to avoid overflow for 32‐bit floating point numbers used for the DL algorithms.

Hyperparameter settings used for training all CNNs included 200 training epochs without early stopping, a learning rate of $8\times 10^{-4}$, a batch size of 10, and the Adam optimizer. The validation data was used to adjust the learning rate using a learning rate scheduler with a patience of 8. Here, the learning rate was set to decrease by five‐fold if the validation loss did not decrease in the last 8 consecutive epochs, with a minimum learning rate of $6\times 10^{-6}$. The CNNs were trained on an Nvidia RTX A6000 (48 GB) GPU, and the training time for one CNN was about 8 – 11hours for all five folds.

### 2.7 Inference and Data Analysis

The output from the CNNs during inference are synthetic diffusion‐weighted images and estimated parameter maps. Synthetic images were subsequently averaged over the three diffusion encoding directions and the five cross‐validation folds (in the given order). From these images, ADC maps were computed for b‐values of 100 and $1000\;s/{\text{mm}}^{2}$. The clinical images were also averaged over all diffusion directions before ADC calculation at the same b‐values. Moreover, an ADC (2/21b ADC) with the images from the 21b scan was calculated in the same manner as for the clinical ADC. Geometric averaging was applied over the three diffusion encoding directions, while arithmetic averaging was employed for averaging over the five folds.

The MRI studies of patients were evaluated by at least two radiologists with clinical expertise in prostate MRI diagnosis. Evaluations were done through consensus and provided a PI‐RADSv2.1 score with lesion location. Regions of interest (ROIs) within the prostate were obtained through manual segmentation by a radiology resident (F.L.) with 5 years of experience. The peripheral zone (PZ) and the transition zone (TZ) of the prostate were drawn for all patients in the test set, based on the clinical images. For cases of PI‐RADS score 3 or higher, regions with suspected tumors were also segmented.

## 3 Results

### 3.1 Diffusion‐Weighted Images

**FIGURE 2:**
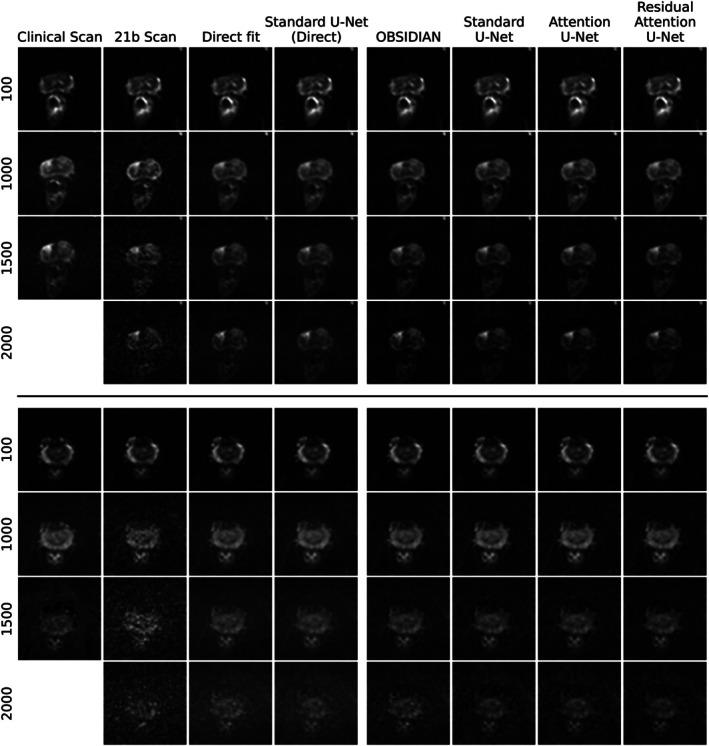
Images zoomed in on prostate gland for one patient from Set 1 (top) and Set 2 (bottom), at different b‐values $\left[{\text{s/mm}}^{2}\right]$. Images are in all cases averaged over the three diffusion encoding directions (trace‐weighted) and over the five cross‐validation folds for the CNNs. The patient from Set 2 had no pathology, whereas the patient from Set 1 was diagnosed with lesions in the transition zone (TZ) and peripheral zone (PZ) with PI‐RADS scores of 5 and 4, respectively, and a Gleason score of 3 + 4. 21b scan images, in comparison to clinical scan images, are acquired without signal averaging, while the clinical image from Set 2 at $b=1500\;{\text{s/mm}}^{2}$ is obtained by means of computed DWI. Images provided by OBSIDIAN, which is based on iterative conventional fitting, and the CNNs employ the biexponential function model. Images generated by the CNNs are slightly smoother than the OBSIDIAN images, particularly at higher b‐values. Images generated with the kurtosis and gamma distribution model function are shown in Figures S1 and S2. Corresponding images showing the full FOV are found in Figures S3, S4 and S5.

Diffusion‐weighted images measured or computed by various implemented modalities are shown in Figure 2, zoomed in on the prostate gland for b‐values of 100, 1000, 1500, and $2000\;s/{\text{mm}}^{2}$ in the case of the biexponential model function. No apparent differences between modalities are observed at $b=100\;s/{\text{mm}}^{2}$. For images with higher diffusion weighting ($b\geq 1000\;s/{\text{mm}}^{2}$), the SNR improvement compared to the 21b scan images is evident. For the 21b images, the prostate becomes gradually indistinguishable as SNR decreases at higher b‐values, whereas Direct fit, OBSIDIAN, and the different CNNs maintain its visibility with reduced noise. Rician bias correction, not employed in the Direct approaches, manifests in lower signal intensities in the tissue around the prostate, resulting in improved contrast. Images generated by the CNNs generally appear smoother in comparison to images generated by OBSIDIAN. Clinical images $b=1500\;s/{\text{mm}}^{2}$ for the patient from Set 2 are generated by means of computed DWI, which appear sharper than the reconstructed images; however, also resulting in diminishing signal around the prostate. The resulting images with kurtosis and gamma distribution as signal models were similar in appearance and are shown in Figures S1 and S2, while versions showing the full FOV are found in Figures S3, S4, and S5.

### 3.2 Generated ADC Maps and Estimated ADC

**FIGURE 3:**
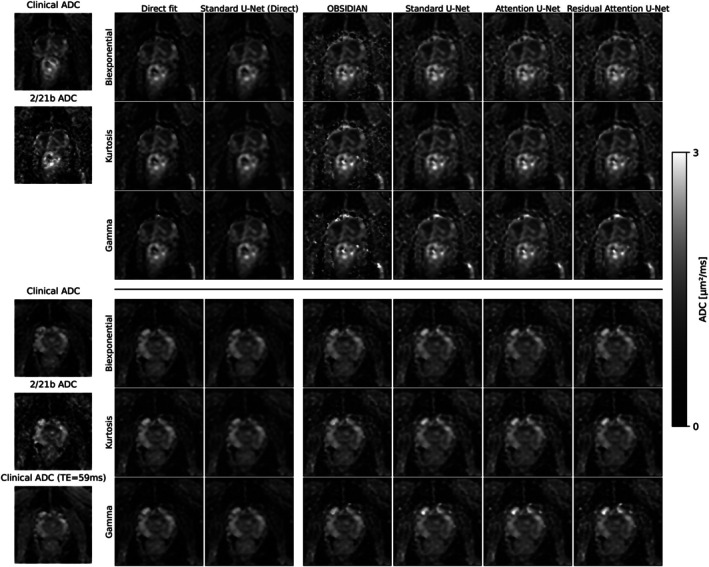
ADC maps of two patients (same as in Figure 2) computed with $b_{100}$‐ and $b_{1000}$‐images derived from the clinical scan, the 21b scan without averaging (labeled as 2/21b ADC) and from denoised images synthesized by direct fitting, OBSIDIAN and the different CNNs. ADC maps from direct fitting, OBSIDIAN, and the CNNs are labeled according to the signal model applied. For the Set 1 patient (top), ADC maps generated by the CNNs do not exhibit the artifactual noise around the prostate gland that is observed with OBSIDIAN for all model functions investigated. The clinical ADC map with TE=59ms is from the standard clinical protocol in Set 2 in comparison to the additional clinical DWI sequence, which had a TE of 74ms equivalent to the 21b sequence. ADC maps from the CNNs were averaged across all folds from five‐fold cross‐validation.

As shown in Figure 3, ADC maps computed from CNN derived diffusion‐weighted images at b‐values of 100 and $1000\;s/{\text{mm}}^{2}$ appear less noisy compared to OBSIDIAN for Set 1, but exhibit similar intensity distribution and preserve anatomical structures for both Set 1 and 2. Also, their visual quality is comparable to the clinical ADC maps and the Direct approaches, also evident in Figure S6, where the full FOV is shown. Higher ADC values due to Rician bias correction are apparent, particularly outside the prostate. There are only subtle differences in the overall appearance between the CNNs across all signal models.

**FIGURE 4:**
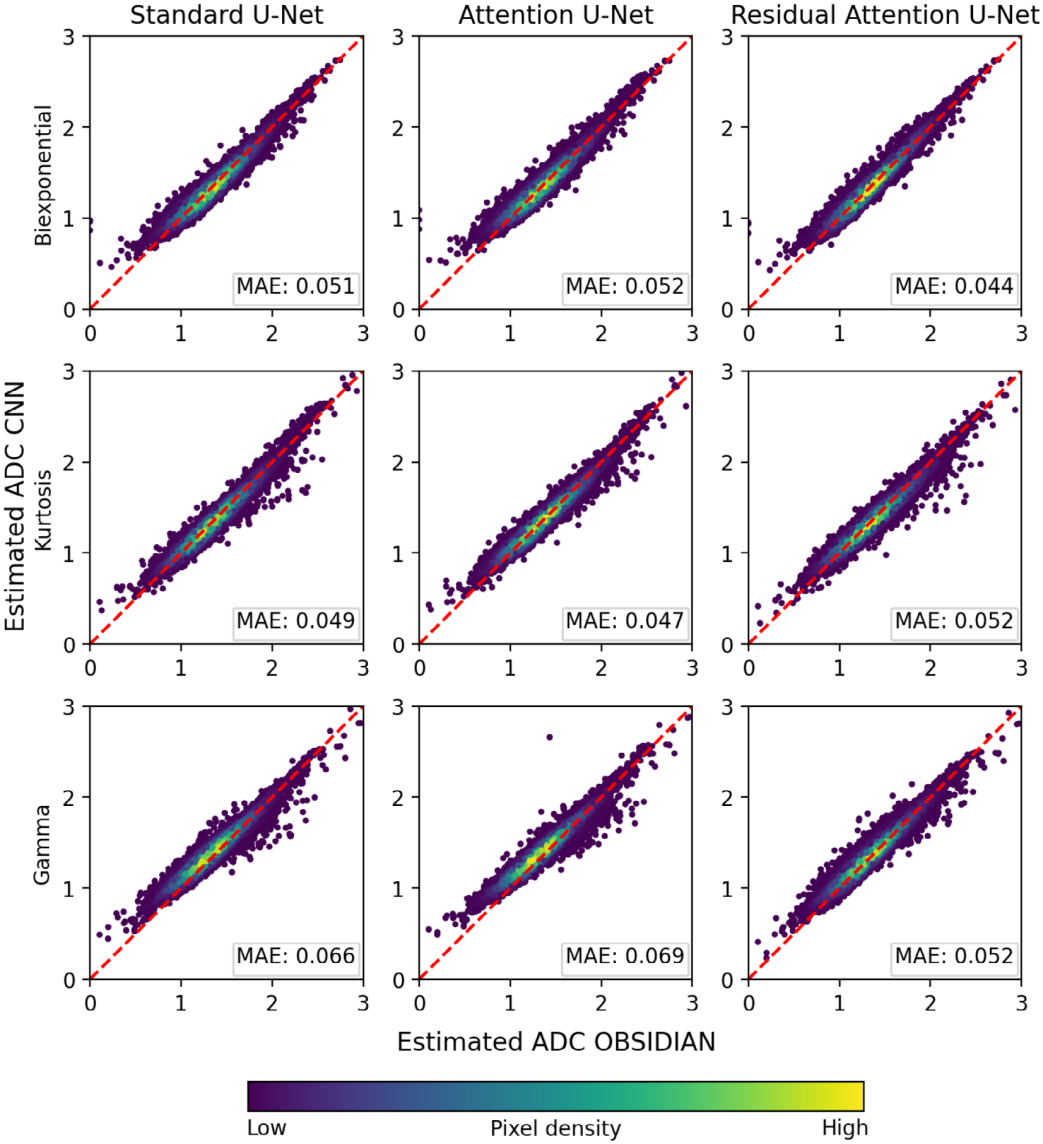
Scatter plots of estimated ADC [$\mu m^{2}/\text{ms}$] by the different methods with respect to OBSIDIAN. The comparison is made in their distribution of ADC pixel values in the healthy TZ of the prostate across all four test patients, with the OBSIDIAN‐ and DL‐estimated ADC represented by the x‐ and y‐axis, respectively. The red identity line indicates perfect agreement between the estimated ADC and OBSIDIAN, where deviations to OBSIDIAN are evaluated as mean absolute error (MAE). Residual Attention U‐Net has the lowest MAE for biexponential and gamma distribution model functions, while for the kurtosis model, the MAE is lowest for the Attention U‐Net.

Averaged ADC values for different prostate ROIs are presented in Table 3. For healthy PZ and TZ, the estimated ADC values by the Rician‐bias‐aware CNNs are close to the OBSIDIAN estimates. On the other hand, the direct approaches result in lower ADC values with estimations close to the clinical ADC for healthy TZ but an underestimation for healthy PZ. For lesions, there is an overestimation of the ADC for the Rician‐bias‐aware CNNs with respect to OBSIDIAN in all cases except for the TZ lesion (Lesion TZ) and Residual Attention U‐Net. In contrast, the direct fit network overestimates lesion ADC in all cases. Figure 4 illustrates the distribution of ADC pixel values across the four test patients in healthy TZ. Lowest mean absolute error (MAE) to OBSIDIAN was observed with the Residual Attention U‐Net for biexponential and gamma distribution model functions and the Attention U‐Net for kurtosis.

**TABLE 3:** ADC estimates [$\mu m^{2}/\text{ms}$] for different methods and higher‐order model functions.

Method	Model	Healthy PZ (23.57 ± 1.13; 4)	Healthy TZ (13.95 ± 1.43; 4)	Lesion PZ(10.67; 1)	Lesion TZ(12.66 ± 1.80; 2)
Clinical	Monoexponential	1.60 ± 0.09	1.28 ± 0.04	0.68	0.85 ± 0.13
2/21b	Monoexponential	1.61 ± 0.08	1.32 ± 0.05	0.71	0.88 ± 0.06
OBSIDIAN	Biexponential	1.61 ± 0.08	1.34 ± 0.04	0.83	0.89 ± 0.08
Direct fit	Biexponential	1.56 ± 0.08	1.27 ± 0.04	0.80	0.87 ± 0.07
Standard U‐Net(Direct)	Biexponential	1.55 ± 0.07	1.27 ± 0.04	0.96	0.93 ± 0.05
Standard U‐Net	Biexponential	1.59 ± 0.08	1.33 ± 0.05	0.92	0.91 ± 0.07
Attention U‐Net	Biexponential	1.59 ± 0.07	1.34 ± 0.05	0.95	0.93 ± 0.07
Residual Attention U‐Net	Biexponential	1.60 ± 0.08	1.35 ± 0.05	0.87	0.89 ± 0.08
OBSIDIAN	Kurtosis	1.61 ± 0.08	1.34 ± 0.04	0.82	0.88 ± 0.08
Direct fit	Kurtosis	1.57 ± 0.08	1.27 ± 0.05	0.80	0.86 ± 0.07
Standard U‐Net(Direct)	Kurtosis	1.56 ± 0.07	1.26 ± 0.04	0.90	0.88 ± 0.07
Standard U‐Net	Kurtosis	1.59 ± 0.08	1.33 ± 0.05	0.89	0.89 ± 0.07
Attention U‐Net	Kurtosis	1.59 ± 0.08	1.35 ± 0.04	0.89	0.91 ± 0.08
Residual Attention U‐Net	Kurtosis	1.58 ± 0.07	1.33 ± 0.04	0.87	0.90 ± 0.08
Residual Attention U‐Net (Noise‐embedded)	Kurtosis	1.59 ± 0.08	1.34 ± 0.04	0.91	0.91 ± 0.07
OBSIDIAN	Gamma	1.59 ± 0.08	1.32 ± 0.04	0.82	0.88 ± 0.08
Direct fit	Gamma	1.54 ± 0.07	1.25 ± 0.04	0.79	0.86 ± 0.07
Standard U‐Net(Direct)	Gamma	1.58 ± 0.07	1.30 ± 0.04	0.97	0.96 ± 0.06
Standard U‐Net	Gamma	1.61 ± 0.07	1.37 ± 0.05	0.99	0.97 ± 0.08
Attention U‐Net	Gamma	1.62 ± 0.07	1.38 ± 0.04	0.98	0.98 ± 0.07
Residual Attention U‐Net	Gamma	1.58 ± 0.07	1.34 ± 0.05	0.93	0.92 ± 0.08

*Note*: ADC values were estimated from test set images and averaged within each region and across the test patients. Average SNR with standard deviation across the test patients and number of cases is given for each region in parentheses. ADC was calculated from $b_{100}$‐ and $b_{1000}$‐images. No significant difference was observed between OBSIDIAN and other methods (p ≤ 0.05, *t*‐test).

*Abbreviations*: ADC, Apparent diffusion coefficient [$\mu m^{2}/\text{ms}$]; OBSIDIAN, optimized bias and signal inference in diffusion image analysis; PZ, peripheral zone; TZ, transition zone.

### 3.3 Kurtosis Parameter Map Estimation

**FIGURE 5:**
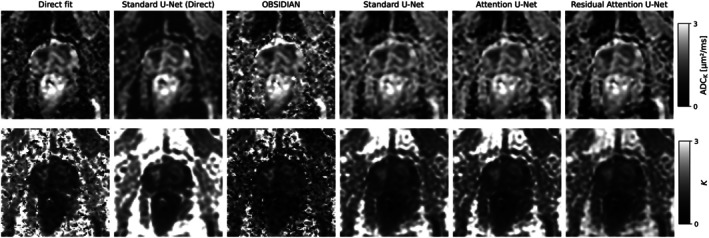
Maps of the kurtosis model parameters ${\text{ADC}}_{K}$ (top) and K (bottom) derived from the different modalities. CNN approaches are considerably less noisy compared to the conventional approaches, Direct fit and OBSIDIAN, particularly for the kurtosis parameter K. Also, the effect of Rician bias correction, not employed by the Direct approaches, is evident. These observations even hold over the full FOV as shown in Figure S10.

The estimated parameter maps for the kurtosis model from our DL framework were less noisy in appearance with better visibility of anatomical structures in comparison to OBSIDIAN and direct fit, as shown in Figure 5. Parameter maps for ${\text{ADC}}_{K}$ and K are similar across the Rician bias‐aware networks. For the standard U‐Net(Direct), some blurriness outside the prostate is observed. For the ROI‐averaged ${\text{ADC}}_{K}$ and K, significant deviation from OBSDIAN is noted for the standard U‐Net (Direct) as shown in Table 4. Generally, the estimated ${\text{ADC}}_{K}$ is lower for the DL approaches compared to OBSIDIAN, except in lesion TZ and PZ, while the estimated K is higher than that for OBSIDIAN except in healthy PZ.

**TABLE 4:** Estimates of kurtosis model parameters ${\text{ADC}}_{K}$ [$\mu m^{2}/\text{ms}$] and K for OBSIDIAN, direct fit and the different CNNs.

Method	Parameter	Healthy PZ	Healthy TZ	Lesion PZ	Lesion TZ
OBSIDIAN	ADC 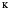	2.08 ± 0.12	1.73 ± 0.07	1.00	1.11 ± 0.08
Direct fit	ADC 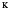	2.09 ± 0.12	1.71 ± 0.07	0.99	1.10 ± 0.08
Standard U‐Net(Direct)	ADC 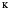	2.04 ± 0.10	1.68 ± 0.07	1.20	1.15 ± 0.10
Standard U‐Net	ADC 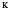	1.95 ± 0.11	1.66 ± 0.06	1.09	1.10 ± 0.09
Attention U‐Net	ADC 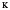	1.96 ± 0.10	1.68 ± 0.06	1.13	1.14 ± 0.09
Residual Attention U‐Net	ADC 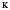	1.95 ± 0.09	1.67 ± 0.06	1.09	1.13 ± 0.11
Residual Attention U‐Net (Noise‐embedded)	ADC 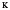	1.95 ± 0.09	1.66 ± 0.06	1.15	1.13 ± 0.08
OBSIDIAN	K	0.57 ± 0.03	0.64 ± 0.04	0.80	0.90 ± 0.13
Direct fit	K	0.64 ± 0.02 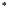	0.77 ± 0.01 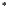	0.89	0.97 ± 0.10
Standard U‐Net (Direct)	K	0.65 ± 0.03 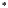	0.82 ± 0.02 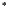	1.14	1.10 ± 0.10
Standard U‐Net	K	0.53 ± 0.03	0.65 ± 0.01	0.93	0.95 ± 0.07
Attention U‐Net	K	0.54 ± 0.03	0.65 ± 0.02	0.99	0.95 ± 0.10
Residual Attention U‐Net	K	0.55 ± 0.03	0.67 ± 0.02	0.99	0.97 ± 0.08
Residual Attention U‐Net (Noise‐embedded)	K	0.53 ± 0.04	0.65 ± 0.02	0.98	0.93 ± 0.10

*Note*: Significant differences from OBSIDIAN (p ≤ 0.05, *t*‐test) are denoted by *.

*Abbreviations*: ADC, Apparent diffusion coefficient [$\mu m^{2}/\text{ms}$]; K, Kurtosis; OBSIDIAN, optimized bias and signal inference in diffusion image analysis; PZ, peripheral zone; TZ, transition zone.

### 3.4 Noise Map as DL Input

**FIGURE 6:**
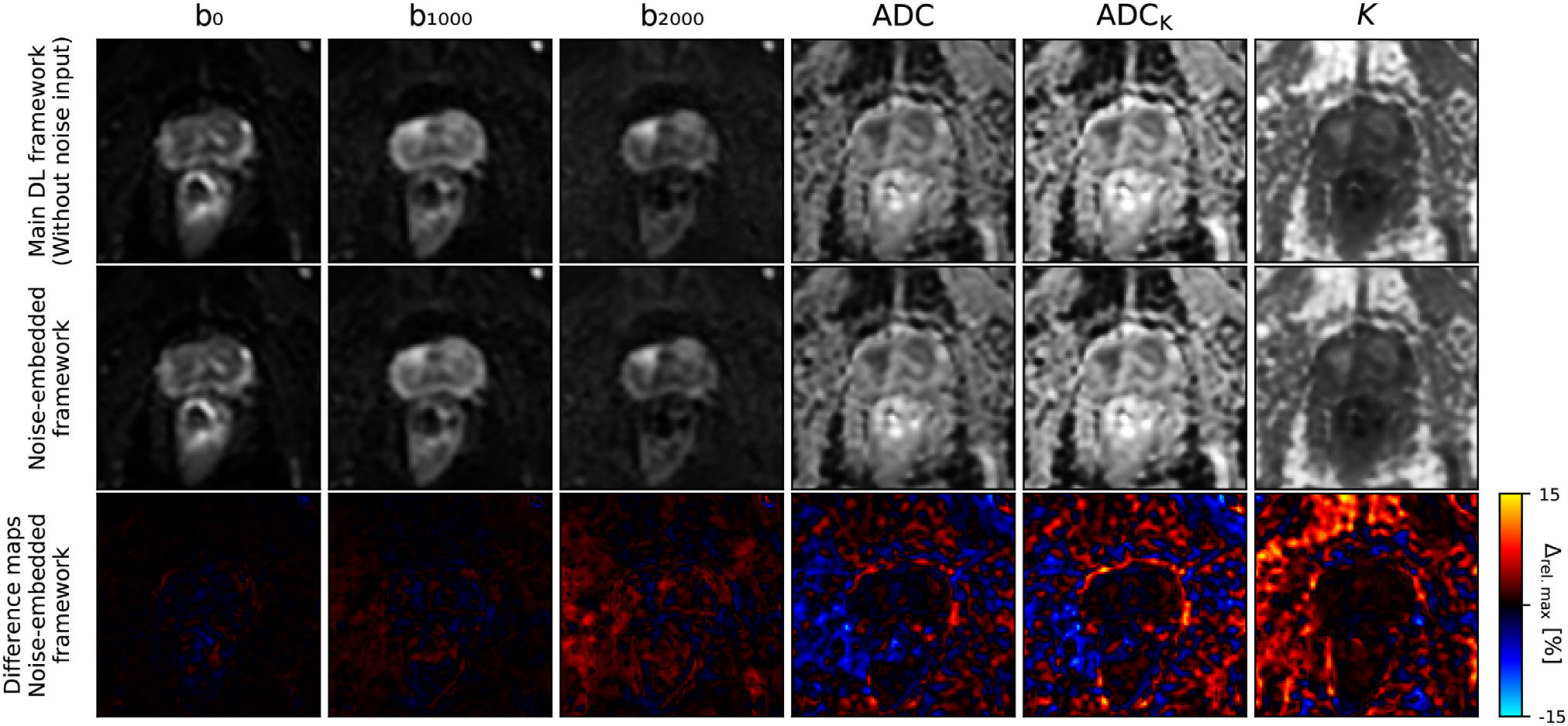
Comparison of diffusion‐weighted images and parameter maps between the main DL framework, without including noise maps as input, and the noise‐embedded framework with noise maps included as input. Adding noise maps as input to the DL algorithm results in very small changes for synthetic image intensities and model parameters within the prostate gland, while changes are more pronounced outside the prostate, as is evident on the corresponding full FOV images shown in Figure S11. The difference maps were computed for each pixel i as the relative difference to the main DL framework normalized by the maximum value for the main DL framework image or map: $\Delta_{\text{rel}}^{\left(i\right)}=(\text{main DL}(i)-\text{noise‐embedded}(i))/(\max(\text{main DL}))$.

Providing the noise maps as DL input (noise‐embedded framework), differences in synthetic image intensities within the prostate with respect to the main framework are small as shown in Figure 6. For $b=2000\;s/{\text{mm}}^{2}$ signal intensity is slightly lower over the full FOV (Figure S11). Model parameter estimations within the prostate are within a few percent of the maximum model parameter value, while there are more pronounced deviations outside the prostate tissue. The estimated ROI‐averaged ADCs were slightly closer to OBSIDIAN than the corresponding approach without noise input for healthy PZ and TZ (Table 3), while there was no consistent trend for the kurtosis model parameters (Table 4).

### 3.5 Ablation Study

The addition of residual connections and attention gates to the standard U‐Net leads to faster convergence and slightly lower training loss in most cases, while only adding attention gates does not have any impact, as seen in Figure S8. A further ablation study is highlighting the importance of concatenation in the U‐Net, as the lack thereof leads to higher training loss (Figure S8) and blurry images (Figure S9).

## 4 Discussion

A self‐supervised training approach was implemented with diffusion‐weighted images, which were acquired without signal averaging, as the input to the CNN models and as the target for synthesized images based on signal models in the loss function. The advantage of self‐supervised training is that it eliminates the need for high SNR reference images that are difficult to acquire. As a consequence, no additional scan time is needed and it furthermore enables continued learning when more patient data is acquired in clinical practice.

The framework described in our previous work allows for denoising, more robust ADC calculation, retrospective application of an arbitrary b‐value acquisition scheme for ADC calculation and holds the potential for application of higher‐order model functions for novel disease biomarkers [18]. The drawback of this conventional model‐based framework, however, is the computationally intensive pixel‐by‐pixel fitting algorithm that renders the framework impractical in a clinical setting. Here, we present a DL driven approach for the framework based on CNNs that reduces the computation time by orders of magnitude, while at the same time delivering synthetic diffusion‐weighted images of comparable quality and improving the visual appearance of model parameter maps that could be used as biomarkers. Fast computation of biomarker maps, as for example ADC maps, is particularly important for large patient cohorts, for example, for disease screening, and in the case of time‐critical diagnostics. In terms of visual quality, the images generated by the different DL structures are very similar to those generated by OBSIDIAN at the clinically relevant b‐values of 1000 and $1500\;s/{\text{mm}}^{2}$. Clinical images and methods without Rician bias correction exhibit higher signal outside of the prostate in accordance with our previous work [18], obscuring anatomical structures to a certain degree. In our opinion, Rician bias correction leads to a more appealing appearance of the diffusion‐weighted images. Furthermore, application of computed DWI can lead to images that are degraded outside the prostate, which can be distracting during diagnostic image evaluation.

With regards to ADC maps, results from the CNNs are of similar visual quality as the clinical ADC maps, even if those were collected at a shorter TE. This indicates that the DL driven approach can compensate for the longer TE resulting from the need to acquire images at higher b‐values. Moreover, the synthetic images at the most relevant diffusion‐weighting for prostate cancer diagnostics ($b=1500\;s/{\text{mm}}^{2}$) are visually more appealing than those generated by computed DWI. Also, for one dataset (Set 1), OBSIDIAN‐generated ADC maps were noise‐inflicted around the prostate tissue in contrast to those from the CNNs. In terms of a quantitative comparison for ROI‐averaged ADC values, differences between higher‐order model functions for the DL approaches are small and comparable to the OBSIDIAN approach. The deviation in ADC of CNNs to OBSIDIAN in healthy TZ correlates with the MAE in the pixelwise comparison, with a lower MAE corresponding to a smaller difference in ADC. Higher deviations are observed for lesions, which might be explained by the low prevalence of tumor tissue in the training data. However, given the low number of lesions in our test data, these results have to be taken with care. Generally, only minor differences were observed among the three higher‐order model functions in line with observations in our previous work [18].

An apparent advantage of the DL‐model from our framework over OBSIDIAN is the smoother appearance of computed model parameter maps, for example, ${\text{ADC}}_{K}$. The DL‐estimated parameter maps have clearer boundaries of the prostate and other structures. This observation might be explained by the inherent difference in the operation of the algorithms. On the one hand, OBSIDIAN is applied pixel‐wise and thus does not capture any spatial context, except for spatial smoothing of the computed noise map, while on the other hand, the DL methods implement convolutional kernels that operate on small image patches and can capture contextual information. Moreover, the added complexity of the Attention och Residual Attention U‐Net structure in comparison to the standard U‐Net implementation does not have a major impact on our results. However, it was observed that the Residual Attention U‐Net converged faster with a slightly lower loss than the other approaches in most cases.

### 4.1 Limitations and Future Perspectives

While the DL‐based framework offers tremendous improvements in computation speed and smoother parameter maps, there are several drawbacks and limitations. First, training of the implemented CNNs is time‐consuming and needs specialized hardware in terms of powerful GPUs (about 8–11h on an Nvidia A6000 GPU). Approaches using simpler structures such as multilayer perceptrons could decrease the computational burden, but at the cost that information on the spatial image context is not considered [43]. Also, early stopping based on observations of the improvement in training loss could be implemented to reduce training time. However, we found that this approach terminated network training too early, leading to inferior visual and quantitative results.

Second, noise maps are generated by a separate algorithm (OBSIDIAN), making the whole training procedure more complex and time‐consuming. As noise maps are often generated in the context of multi‐coil reconstruction, as for example in SENSE [44], they might be directly exported from the scanner, if access is granted by the manufacturer. Also, generating the noise maps with a different algorithm could be contemplated. In our previous work, we found that the commonly employed methods of MP‐PCA [14] were significantly underestimating noise for our 21‐b data with 3 diffusion directions [16]. Certain commonly employed denoising algorithms, such as, for example, Noise2Noise [45] or Noise2Self [46], do not estimate the noise standard deviation and are thus not applicable. Moreover, techniques based on the complex signal are promising but are complicated as they need access to the reconstruction pipeline and phase correction needs to be performed [47]. Furthermore, predicting the noise distribution as an additional model parameter for our DL implementations was tested, but resulted in inaccurate noise maps. This is in line with similar efforts for more conventional methods [17].

Third, for a more thorough statistical analysis, a larger number of test cases would be needed for our study. Furthermore, one would like to acquire a gold standard based on repeated acquisition as outlined by Manzano Patron et al. [48]. However, repeating a 4‐minute sequence multiple times leads to long acquisition times that are hard to accommodate in the clinical workflow and might cause distress for the patient. Even if that problem could be overcome, confounding errors, such as signal drift [49] and the fact that averaging a Rician distributed signal does not eliminate the inherent signal bias [16] need to be addressed.

Fourth, generalizability is a common issue for DL implementations. A neural network trained on a specific patient cohort, body region, and MR scanner settings is generally bound to be applied in a scenario with little variation in respect to the training case or otherwise needs to be fully or at least partially retrained (transfer learning) on training data reflecting this change. In particular, our algorithms are trained for specific b‐values and cannot process data where b‐values are omitted, added, or changed. Acquisition resolution can, in principle, be altered if resampling to the same effective voxel size is applied before inference. However, using, for example, a smaller acquisition voxel size results in a narrower point spread function if other acquisition parameters, for example, TE, can be kept constant. This in turn, means that each 3×3 image patch seen by the kernel of our CNN implementations has less smoothing applied, which can have an effect on CNN prediction. On the other hand, variations in the FOV while keeping other acquisition parameters constant effectively result in a shift of the image borders and are expected to result in very small changes in the CNN output within the prostate if FOV variations are minor and if the image normalization factor is not heavily changed. However, changing resolution or FOV usually requires changes in TE affecting image contrast and underlying model parameters, which might ultimately lead to out‐of‐distribution predictions. In the same sense, using a network trained on prostate data for brain images, even if acquisition parameters are identical, is not advised, as object geometry and underlying parameter distribution are different. Conventional approaches, such as, for example, OBSIDIAN, are not at all or much less affected by such modifications. In principle, this problem could be solved by training on a dataset with a variety of acquisition parameters, body regions, and patient cohorts. However, this would require large amounts of data, and performance on a specific case might be inferior to a network specialized in a certain scenario. Moreover, it shall be pointed out that for many routine scans, as well as for the potential application of prostate cancer screening, scanner protocols stay more or less fixed for a number of patients far exceeding the number of cases needed for training. This means that a trained network could be used quite efficiently.

Fifth, we opted to have similar TEs for the clinical and 21b sequences in Set 1 and Set 2. For Set 2 this resulted in an increase of TE for the clinical sequence from 59ms to 74ms corresponding to a decrease in SNR by 9% for normal and 19% for cancerous tissue when using $T_{2}$ values of 170ms and 85ms, respectively ($T_{2}$ values taken from [50]). Implementing an approach where the overplus option for the 21b sequence is activated could allow for a slightly more realistic comparison of clinical and DL based images and biomarker maps. Moreover, one could consider only acquiring b‐values up to $1500\;s/{\text{mm}}^{2}$ for the 21b sequence to further reduce TE. The loss in image quality and robustness for our DL based synthetic images and biomarker maps is potentially as small as in our previous work for conventional model fitting [18]. However, it should also be taken into account that TEs around 75ms are more realistic on conventional scanners limited to gradient amplitudes of around 45mT/m even with some form of gradient overplus activated. As such, the CNNs trained in this work could be directly applied to data acquired on these scanners.

Sixth, our DL implementation indirectly learns to account for the noise distribution in the optimization process during training, but does not take the noise map as an input parameter. Should the noise distribution for an unknown dataset be significantly different from training cases, for example, if a completely different coil configuration is used or the prostate is not in the center of the image, then correct parameter estimation might be inferior compared to OBSIDIAN. Results for the noise‐embedded framework indicate that the added information for the CNN might have an impact on the model output, but needs to be further explored for larger datasets. Providing the OBSIDIAN noise map as an additional direct input, however, would mitigate the speed advantage for inference, as the data would have to be preprocessed with OBSIDIAN. Providing noise maps by other means would again be a solution that can be explored. With regards to inter‐scanner generalizability, data was processed from two different scanners to introduce some data heterogeneity, although the scanners are from the same manufacturer, and use the same field strength and gradient performance.

Finally, even though the higher order models applied in the present study capture some underlying physics, even more complex signal models could be tested, as for example models based on Verdict [31, 51].

## 5 Conclusions

We have successfully implemented a DL approach for the framework presented in our previous work [18] where a large range of b‐values are processed for reproducible model fitting and ADC measurements in a clinical setting. The drastically reduced computation time makes the framework feasible for screening cohorts and in time‐critical applications. Synthesized images and parameter maps are of similar or even better quality than in the previous approach, which employed iterative conventional fitting, for most of the combinations of signal model function and CNNs, with the results from the Residual Attention U‐Net being visually most appealing. All CNNs yield reasonable ADC estimates in the different prostate regions for our testing data. Still, our method may be further refined by employing larger datasets in order to achieve better generalizability and increased robustness.

## Funding

This work was supported by the Swedish Cancer Society, Swedish Prostate Cancer Association, Assar Gabrielsson Foundation (BGR23‐06), Stiftelsen Jubileumsklinikens Forskningsfond mot Cancer (2024:583). Medical Education and Research in a clinical setting (ALFGBG 932648), the Swedish Research Council and the Swedish Cancer Society.

## Disclosure

The authors have nothing to report.

## Supporting information




Data S1.
**Figure S1.** Same as Figure 2, but with kurtosis model.
**Figure S2.** Same as Figure 2, but with gamma distribution model.
**Figure S3.** Same as Figure 2, but showing the full FOV.
**Figure S4.** Same as Figure S1, but showing the full FOV.
**Figure S5.** Same as Figure S2, but showing the full FOV.
**Figure S6.** Same as Figure 3, but showing the full FOV.
**Figure S7.** Architectures of CNNs used in this study. Here, the U‐Net model A) was used with additional convolution layers in the beginning. Only a section with the modified components is shown for the Attention U‐Net B) and Residual Attention U‐Net C). The final output is N signal parameter maps where, as an example, N=7 for the 3D kurtosis model.
**Figure S8.** Loss curves for different AI models evaluated in this study. For kurtosis, an additional U‐Net (Ablated) model is included, where the concatenation is removed. Residual Attention U‐Net shows consistently faster convergence across the function models, and U‐Net (Ablated) exhibits a notably higher loss compared to the standard U‐Net.
**Figure S9.** Comparison between a conventional U‐Net model and an ablated U‐Net, in which the concatenation is removed. A clear loss of image details is observed, highlighting the importance of skip connections.
**Figure S10.** Same as Figure 5, but showing the full FOV.
**Figure S11.** Same as Figure 6, but showing the full FOV.

## Data Availability

The authors have nothing to report.
