# Supplementary material for: Self‐Supervised Deep Learning Framework for Rician Distribution Based Denoising and Modeling of Multi‐b Prostate Diffusion MRI
Source: Magn Reson Med. 2026 Apr 19;96(2):928–42. doi: 10.1002/mrm.70373 (PMC13269250; doi:10.1002/mrm.70373)
Supplement: Supplementary file 1 — Data S1. Figure S1. Same as Figure 2, but with kurtosis model. Figure S2. Same as Figure 2, but with gamma distribution model. Figure S3. Same as Figure 2, but showing the full FOV. Figure S4. Same as Figure S1, but showing the full FOV. Figure S5. Same as Figure S2, but showing the full FOV. Figure S6. Same as Figure 3, but showing the full FOV. Figure S7. Architectures of CNNs used in this study. Here, the U‐Net model A) was used with additional convolution layers in the beginning. Only a section with the modified components is shown for the Attention U‐Net B) and Residual Attention U‐Net C). The final output is N signal parameter maps where, as an example, N=7 for the 3D kurtosis model. Figure S8. Loss curves for different AI models evaluated in this study. For kurtosis, an additional U‐Net (Ablated) model is included, where the concatenation is removed. Residual Attention U‐Net shows consistently faster convergence across the function models, and U‐Net (Ablated) exhibits a notably higher loss compared to the standard U‐Net. Figure S9. Comparison between a conventional U‐Net model and an ablated U‐Net, in which the concatenation is removed. A clear loss of image details is observed, highlighting the importance of skip connections. Figure S10. Same as Figure 5, but showing the full FOV. Figure S11. Same as Figure 6, but showing the full FOV. [file MRM-96-928-s001.pdf]

Supporting Information

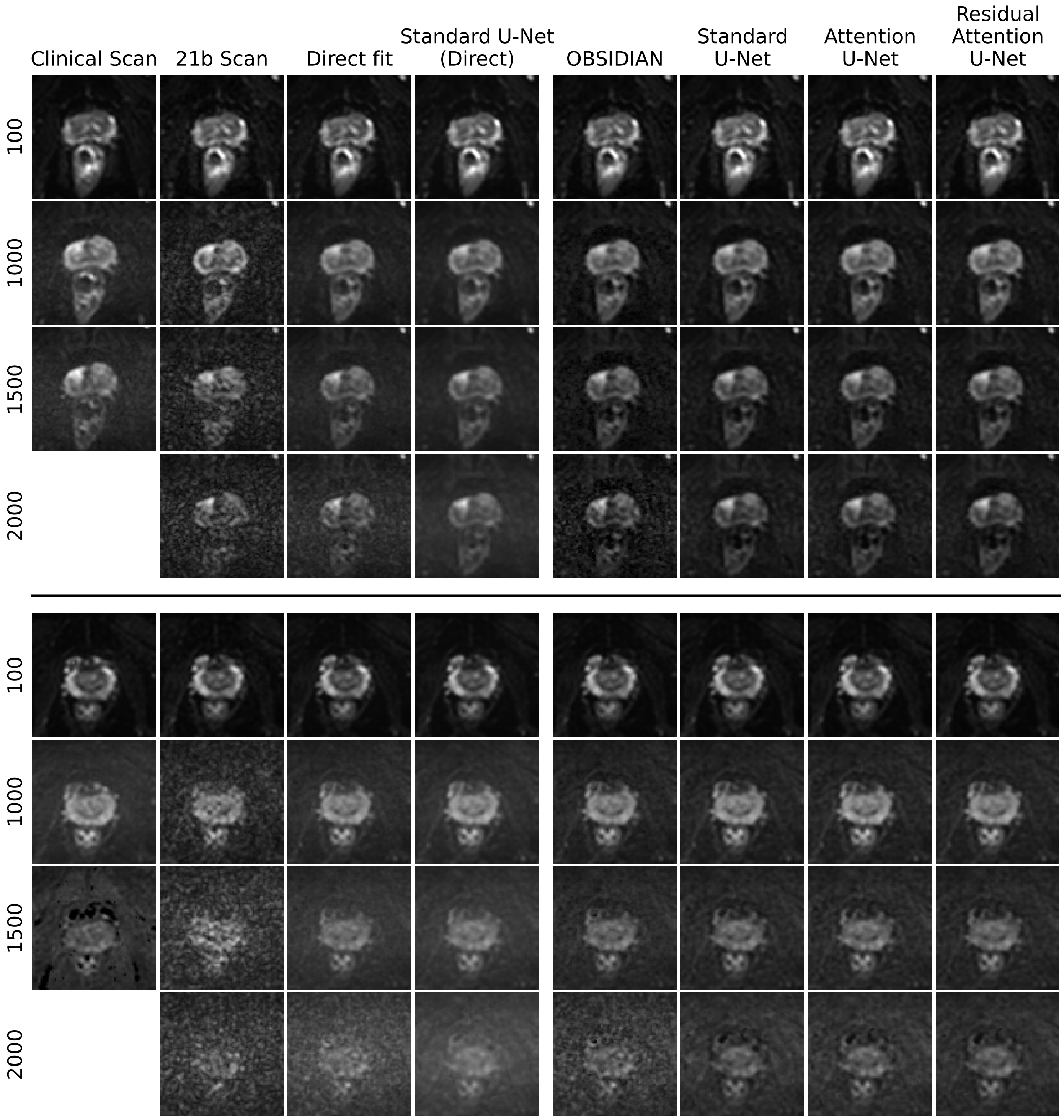

Figure S1: Same as Figure 2 , but with kurtosis model.

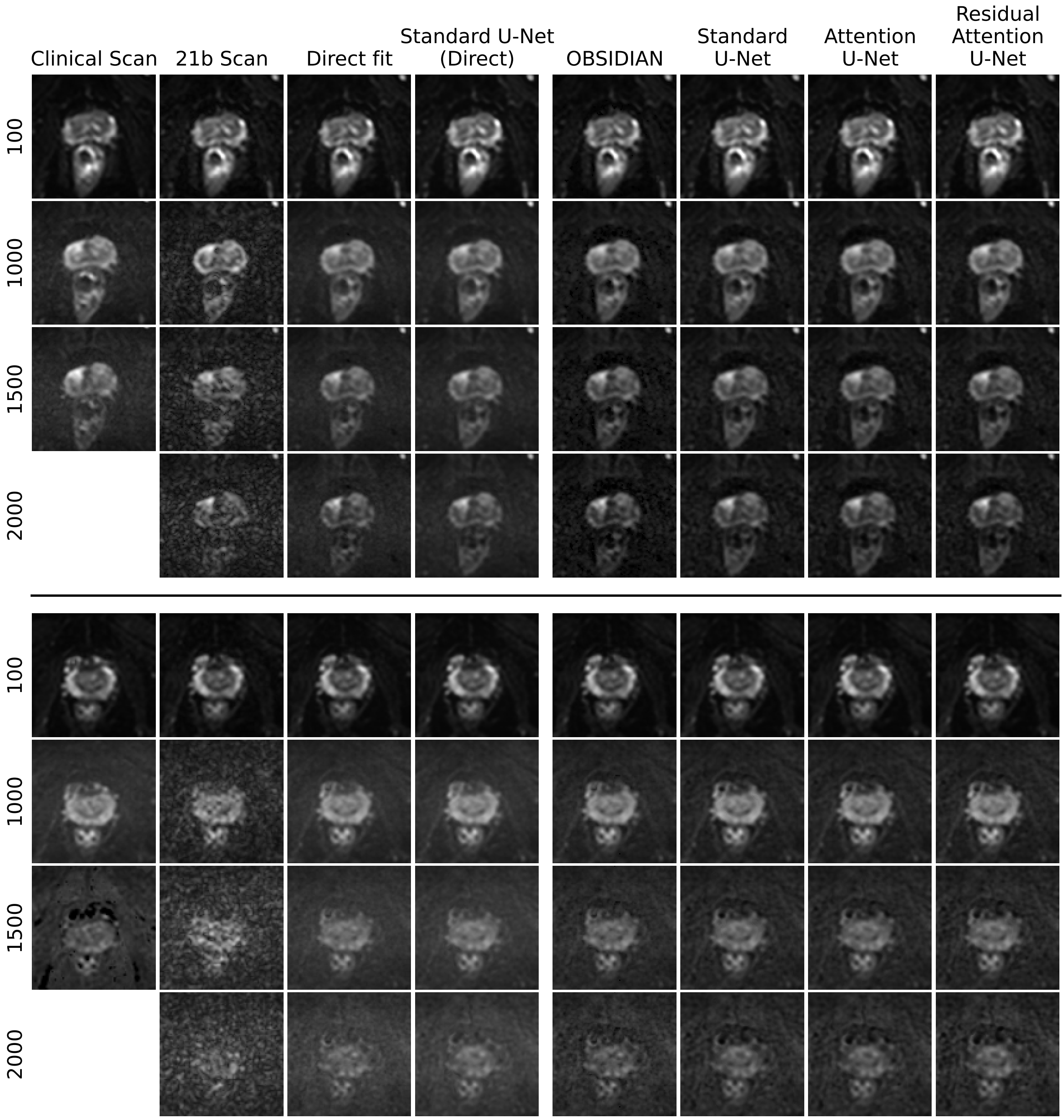

Figure S2: Same as Figure 2 , but with gamma distribution model.

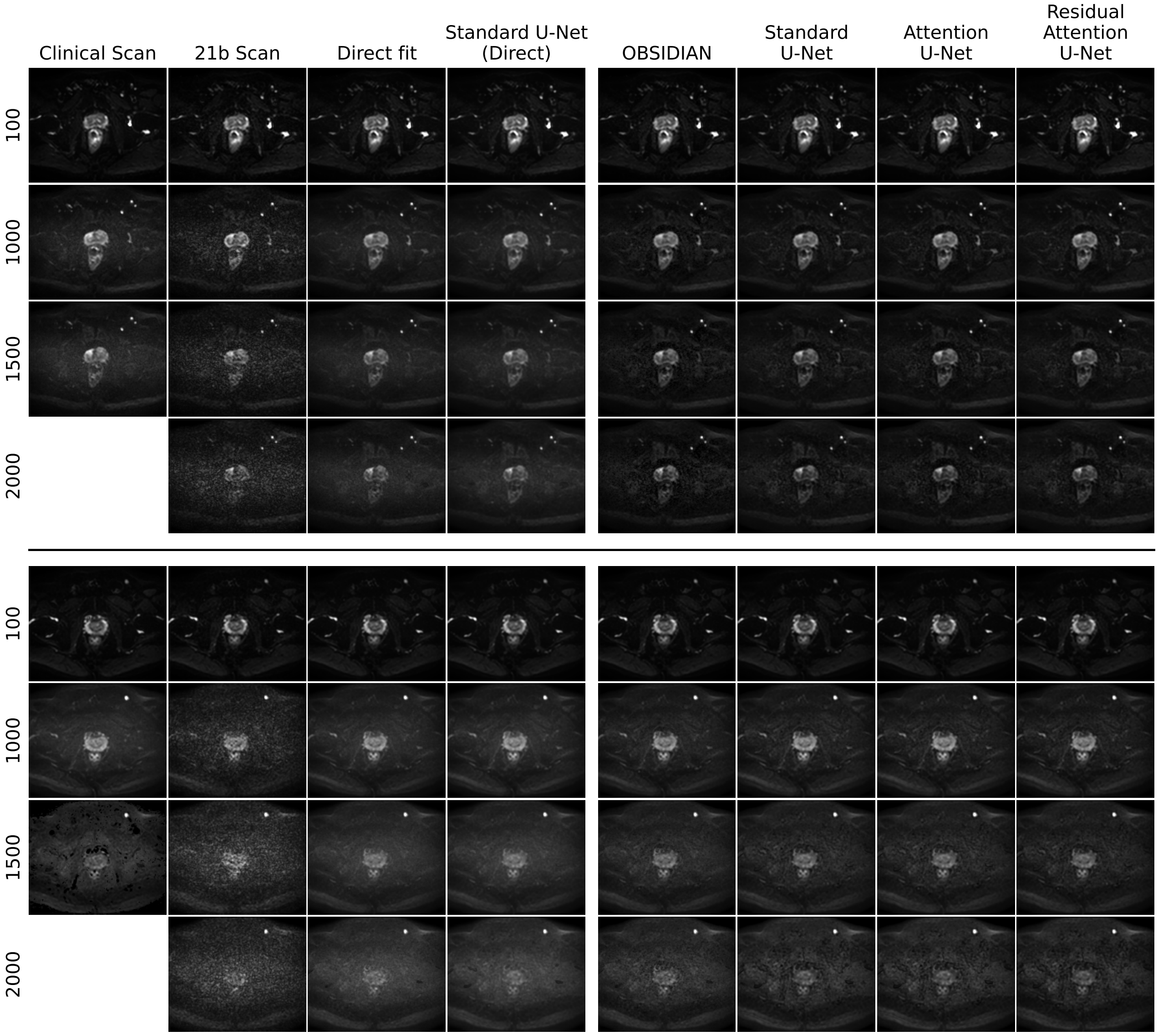

Figure S3: Same as Figure 2 , but showing the full FOV.

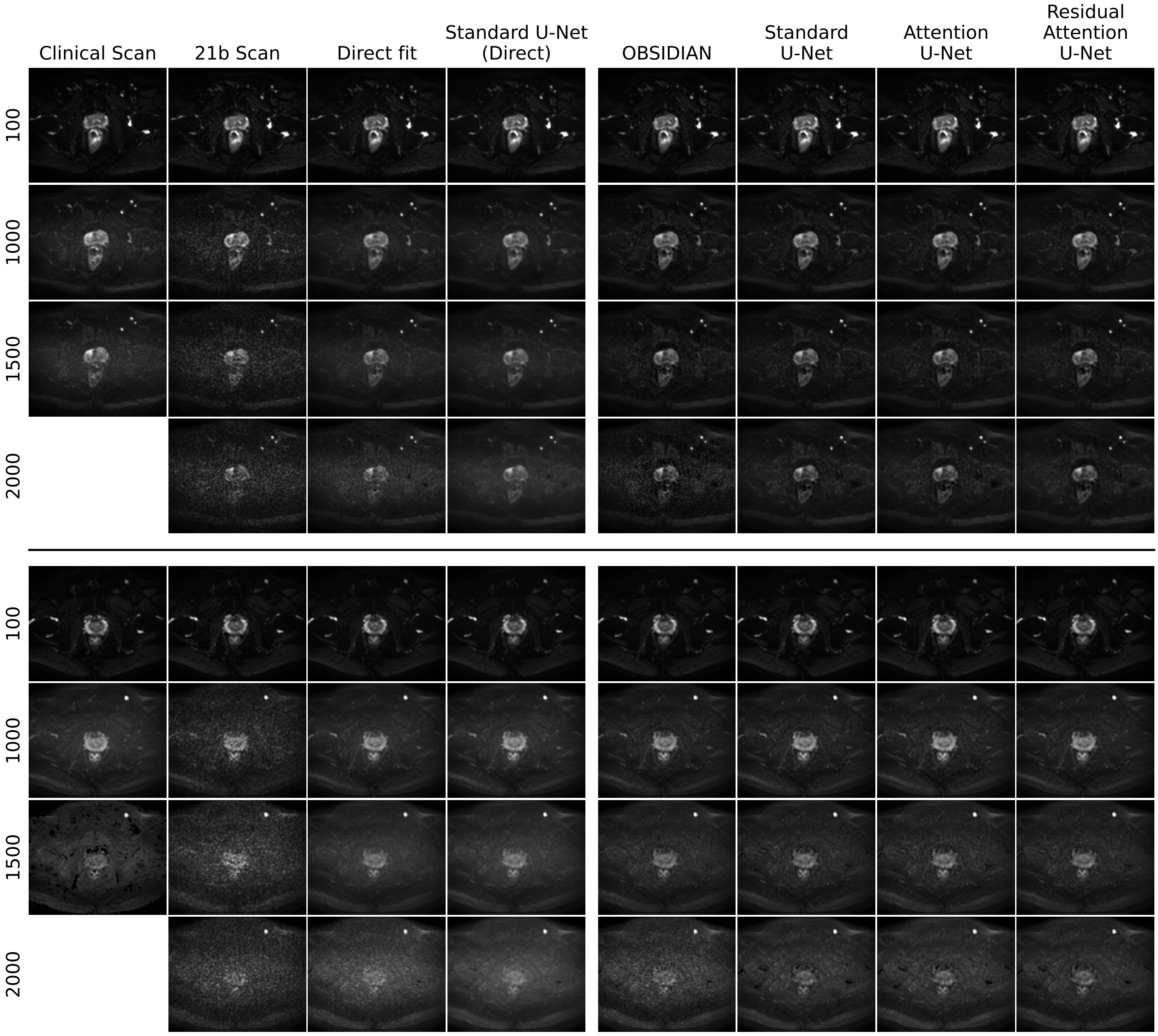

Figure S4: Same as Figure S1, but showing the full FOV.

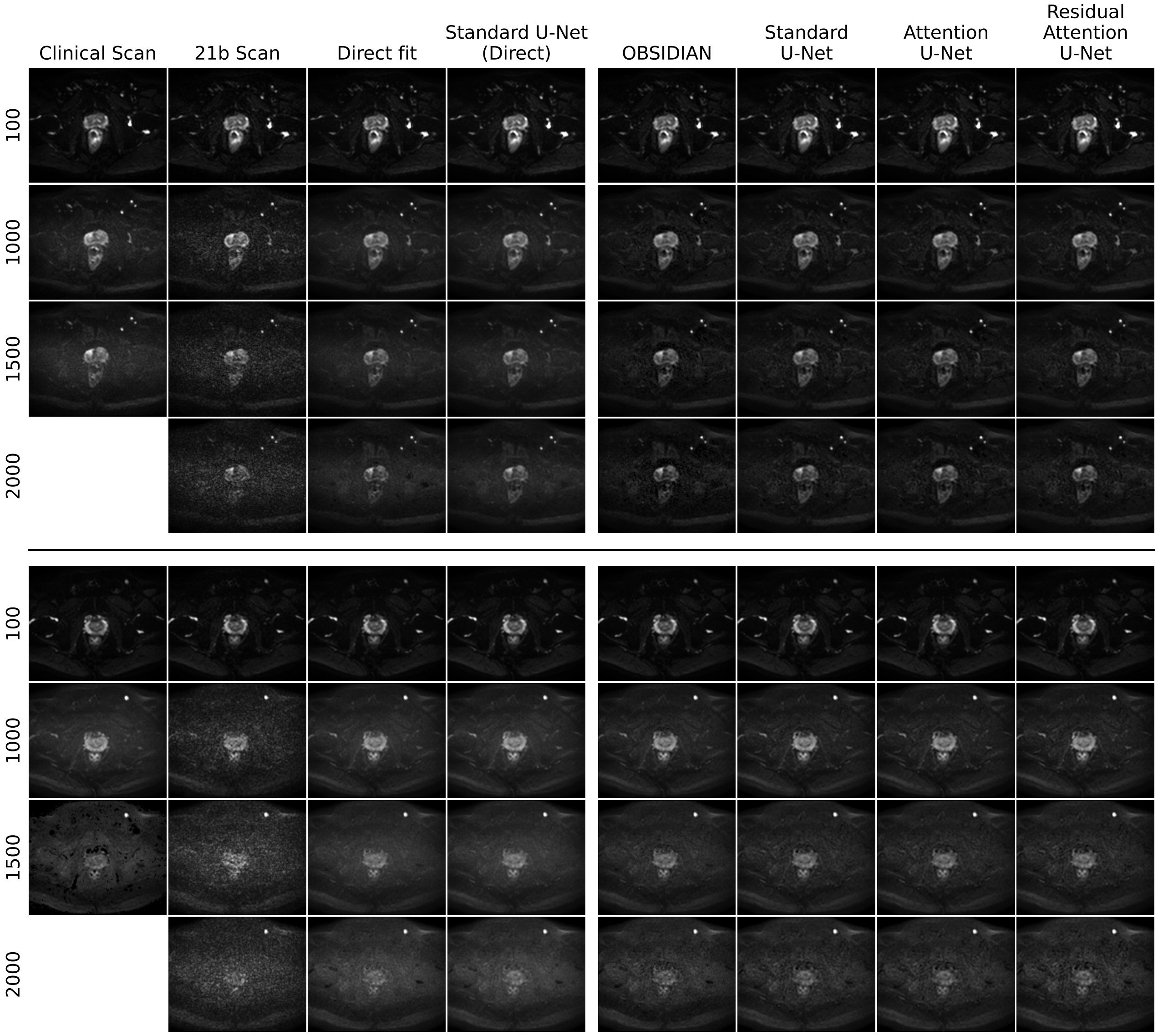

Figure S5: Same as Figure S2, but showing the full FOV.

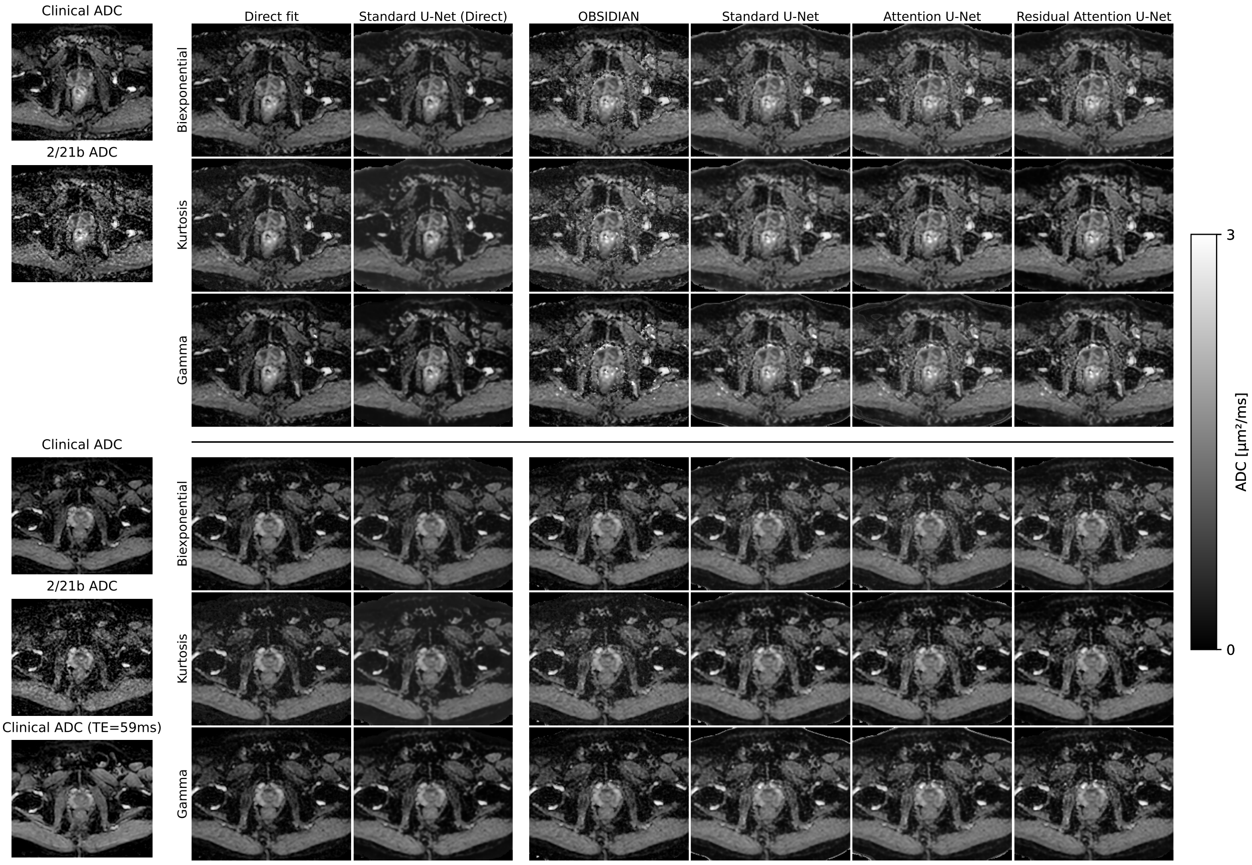

Figure S6: Same as Figure 3 , but showing the full FOV.

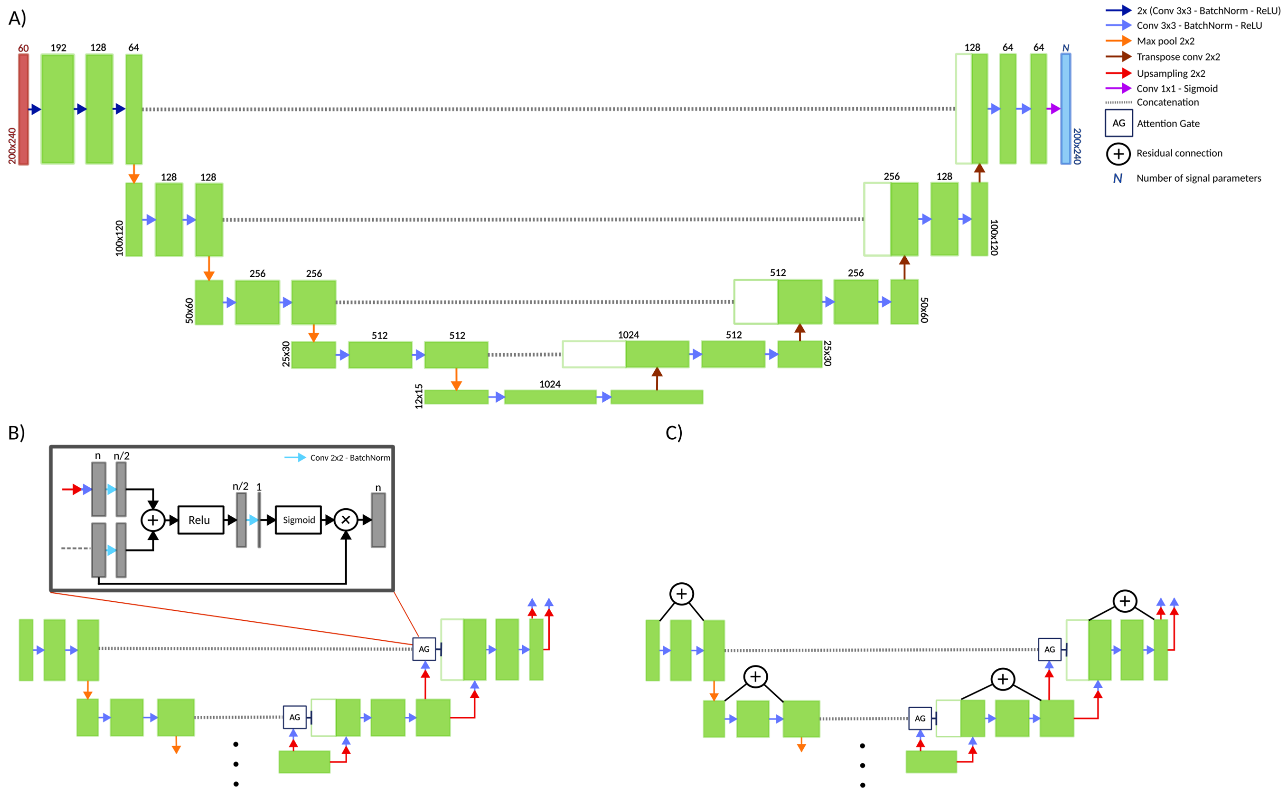

Figure S7: Architectures of CNNs used in this study. Here the U-Net model A) was used with additional convolution layers in the beginning. Only a section with the modified components are shown for the Attention U-Net B) and and Residual Attention U-Net C). The final output is N signal parameter maps where, as an example, $N = 7$ for the 3D kurtosis model.

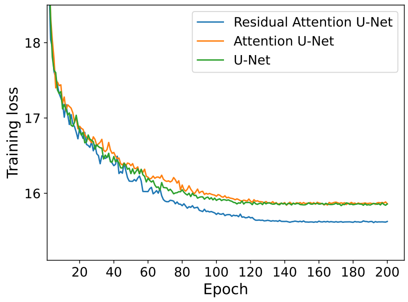

(a) Biexponential

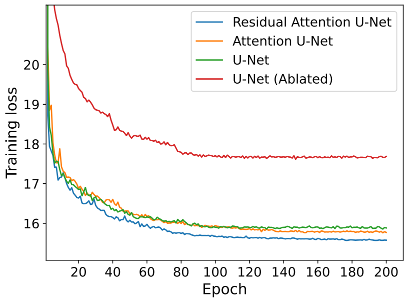

(b) Kurtosis

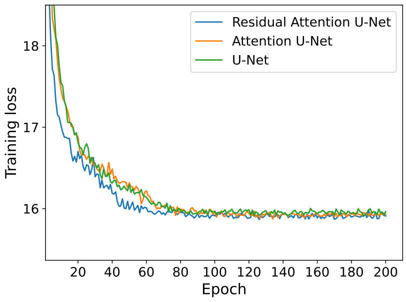

(c) Gamma

Figure S8: Loss curves for different AI models evaluated in this study. For kurtosis, an additional U-Net (Ablated) model is included were the concatenation is removed. Residual Attention U-Net shows consistently faster convergence across the function models and U-Net (Ablated) exhibits a notably higher loss compared to the standard U-Net.

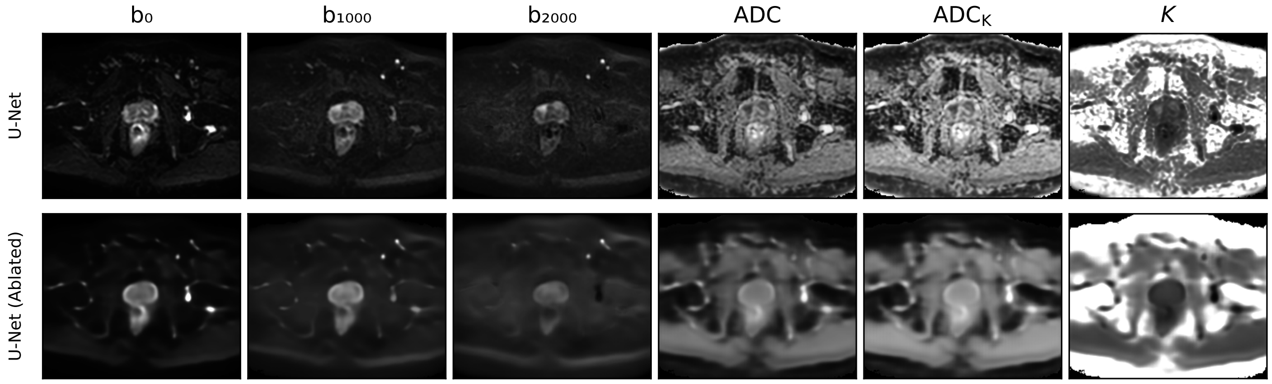

Figure S9: Comparison between conventional U-Net model and an ablated U-Net, in which the concatenation is removed. A clear loss of image details is observed, highlighting the importance of skip connections.

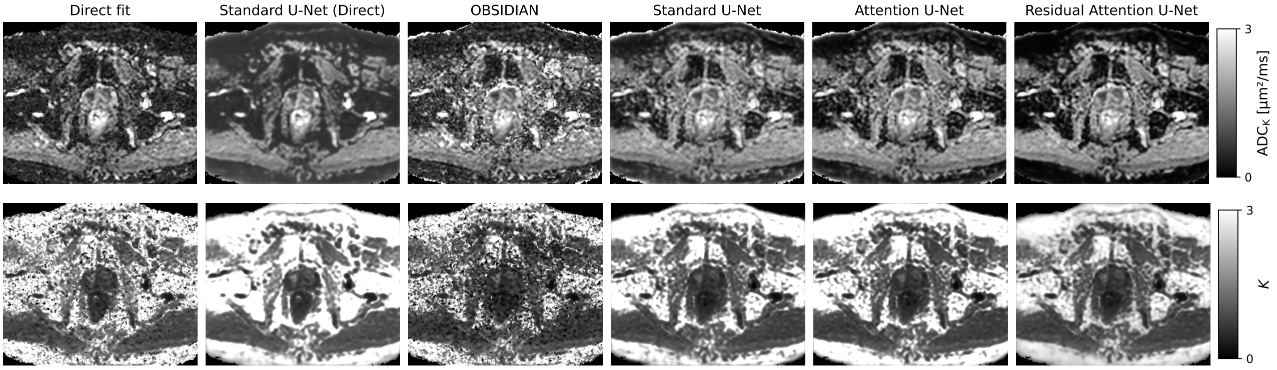

Figure S10: Same as Figure 5 , but showing the full FOV.

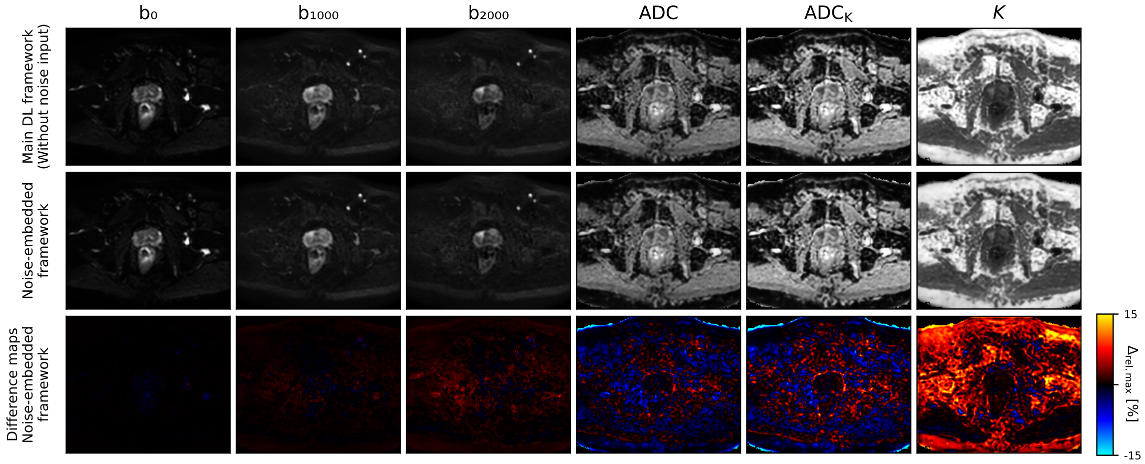

Figure S11: Same as Figure 6 , but showing the full FOV
